# Widespread enlarged perivascular spaces associated with dementia and focal brain dysfunction: case report

**DOI:** 10.1186/s12883-017-0997-9

**Published:** 2017-12-07

**Authors:** Daisuke Taniguchi, Hideki Shimura, Masao Watanabe, Nobutaka Hattori, Takao Urabe

**Affiliations:** 1 Department of Neurology, Juntendo University Urayasu Hospital, 2-1-1 Tomioka, Urayasu, Chiba 279-0021 Japan; 2 Department of Neurology, Juntendo University School of Medicine, 2-1-1 Hongo, Bunkyo-ku, Tokyo, 113-8421 Japan

**Keywords:** Perivascular space, Virchow-Robin space, Dementia, Magnetic resonance imaging

## Abstract

**Background:**

Enlarged perivascular spaces (PVS) are common magnetic resonance imaging (MRI) findings, whereas widespread enlarged PVS are extremely rare. Although most patients with widespread enlarged PVS remain asymptomatic, some develop neurological dysfunctions; however, it remains unclear whether these are the consequence of widespread enlarged PVS.

**Case presentation:**

A 64-year-old female patient developed consciousness disturbance, cognitive dysfunctions, fluent aphasia, agraphia, acalculia, and left-right disorientation after suffering from bronchopneumonia. Brain MRI revealed unusually widespread enlarged PVS predominantly in the left cerebral hemisphere. Following bronchopneumonia treatment, her cognitive dysfunction, fluent aphasia, agraphia, acalculia, and left-right disorientation persisted despite improvement of her general condition. Furthermore, the hypoperfusion area on single photon emission computed tomography and slow wave sites on electroencephalography were consistent with the location of enlarged PVS, indicating that severe enlarged PVS impaired focal brain functions.

**Conclusions:**

This case suggested that widespread enlarged PVS could be a potential cause of neurological deficits. We propose that impaired perivascular circulation due to enlarged PVS might lead to focal brain dysfunction.

## Background

Perivascular spaces (PVS), also known as Virchow-Robin spaces, surround the walls of brain parenchyma penetrating arteries, veins, and venules [1]. Overall, PVS are very common and considered normal findings on magnetic resonance imaging (MRI) of healthy individuals of all ages. These, however, can reach larger sizes, so-called enlarged PVS, especially in the elderly [1]. Widespread enlarged PVS are extremely rare and may signal brain abnormalities [2–13]. Although most patients with widespread enlarged PVS remain asymptomatic, some develop neurological deficits [2–4]. However, it is not clear whether enlarged PVS account for these deficits. Here, we report the case of a patient with widespread enlarged PVS, who has developed cognitive impairments and higher cerebral dysfunction upon contracting bronchopneumonia.

## Case presentation

A 64-year-old female patient was admitted to the emergency unit in our hospital due to progressive alterations of her mental status. Her medical history and that of her family were both unremarkable except that she was reported to have had several small brain cysts in the right posterior lobe while in her 30s. Three days prior to her admission, she developed a fever with upper respiratory tract symptoms, and complained of fatigue. On admission, her vital signs revealed a blood pressure of 180/100 mmHg, heart rate of 120 beats per minute, temperature of 38.0 °C, and oxygen saturation level of 98%. Her blood tests revealed elevated white-blood-cell counts (14,700 cells/μL) and C-reactive protein level (8.2 mg/dL). Her renal and liver functions as well as the levels of serum electrolytes were normal. The neurological examination revealed a Glasgow Coma Scale score of E4V4M6, suggesting mental confusion. She showed temporal disorientation, and developed fluent aphasia, agraphia, acalculia, and left-right disorientation. However, she showed no weakness, ataxia, sensory disturbance, autonomic nervous dysfunction, or signs of meningeal irritation. Cerebrospinal-fluid (CSF) examination revealed normal opening pressure (120 mmH_2_O), cell counts (2/μL, 100% monocytes), and protein levels (20 mg/dL). The CSF bacterial, fungal, and mycobacterium culture, CSF cytology, and cryptococcal antigen in serum tested all negative. Chest computed tomography (CT) revealed bronchopneumonia in the bilateral inferior lobes. We immediately started bronchopneumonia treatment with intravenous administration of ceftriaxone (2 g/day) for 5 days. Two days after admission, the fever subsided, the level of consciousness increased, and the patient became alert. However, her aphasia, agraphia, and acalculia persisted. Five days after admission, the patient was subjected to several neuropsychological examinations. Her Mini-Mental State Examination (MMSE) score was 18/30, suggesting recent memory loss, impaired attention, acalculia, and visual-constructive agnosia. Her total score on Frontal Assessment Battery was 5/18, suggesting frontal lobe dysfunction. Furthermore, the patient scored 14/36 on the Raven’s colored progressive matrices test, which is a non-verbal evaluation of abstract reasoning. Electroencephalography (EEG) revealed slowing in the theta range predominantly in the left hemisphere, without epileptiform discharge. The patient’s general condition continued to recover, and she was discharged 15 days after admission. However, the residual symptoms of recent memory loss, aphasia, agraphia, and acalculia persisted.

**Fig. 1:**
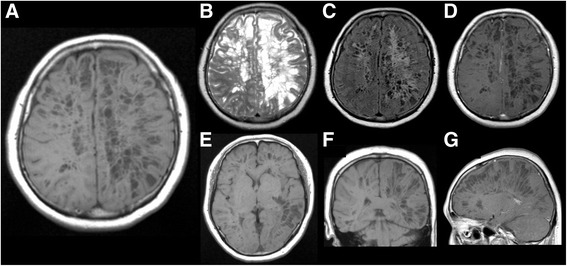
Brain magnetic resonance imaging (MRI) showing multiple, confluent, and oval cystic lesions predominantly in the left cerebral hemisphere on T1 weighted imaging (**a**, **e**, **f**), T2 weighted imaging (**b**), Fluid Attenuated Inversion Recovery imaging (**c**), and gadolinium contrast enhancement imaging (**d**, **g**). Cystic lesions were found in particular in the angular gyrus and frontotemporal lobes (**a**, **e**). They are isointense to CSF (**c)**, surrounded by rim of hyperintensities (**c**), and observed without gadolinium contrast enhancement (**d**, **g**)

**Fig. 2:**
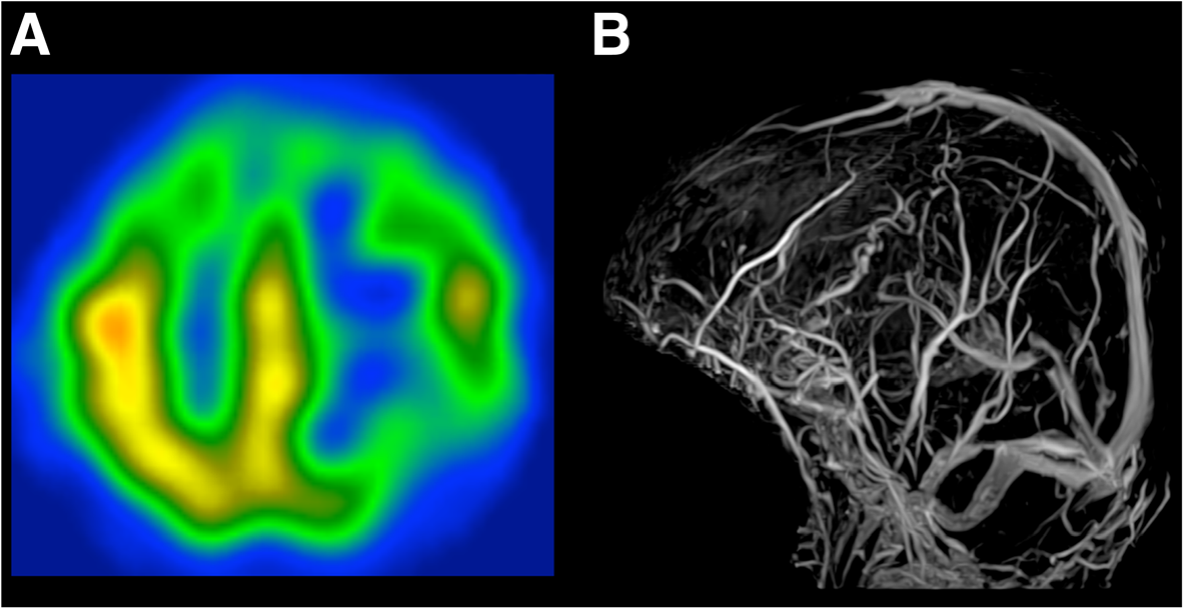
Extended areas of hypoperfusion in the entire left hemisphere and right frontal lobe were observed by p-iodoamphetamine-single photon emission computed tomography (IMP-SPECT) (**a**). Magnetic resonance venography revealed no significant occlusive regions in the cerebral venous system (**b**)

Brain MRI performed on the day of admission demonstrated multiple, confluent, well defined cystic lesions, predominantly in the left cerebral hemisphere (Fig. 1a–g). The cystic lesions were particularly found in the angular gyrus and the frontotemporal lobes (Fig. 1a–g). The Fluid Attenuated Inversion Recovery images revealed that the cystic lesions were isointense to CSF, and surrounded by rim of hyperintensities suggestive of perilesional gliosis (Fig. 1c). The cystic lesions were observed without gadolinium contrast enhancement (Fig. 1d, g). Furthermore, N-isopropyl-[123I] p-iodoamphetamine-single photon emission CT (123I-IMP SPECT) revealed extended areas of hypoperfusion in the entire left hemisphere and right frontal lobe, which may have resulted from the decrease of brain parenchyma volume (Fig. 2a). Magnetic resonance venography revealed no significant occlusive regions in the cerebral venous system (Fig. 2b). After excluding other causes of cystic conditions, a diagnosis of widespread enlarged PVS was made.

Through a two-year follow-up, brain MRI showed no remarkable changes compared with the initial MRI. The cognitive impairments and higher cerebral dysfunctions, such as aphasia, agraphia, and acalculia, did not deteriorate further, but were persistent as evidenced by an MMSE score of 18/30, which was evaluated every 6 months.

## Discussion and conclusions

The MRI features of our patient were consistent with a widespread enlarged PVS [1]. The diagnostic work-up could exclude other causes of cystic condition, such as cystic neoplasm, cryptococcosis, neurocysticercosis, and mucopolysaccharidoses. There were no risk factors for cerebrovascular diseases. The patient was reported to have had several PVS almost 30 years before admission. Furthermore, the appearance of her PVS did not change after a 2-year follow-up. These findings suggested that a widening of PVS might have progressed very slowly and asymptomatically in this case over the years before admission.

Indeed, most cases of widespread enlarged PVS show no neurological symptoms, and present with intact sensory and motor systems evidenced by normal sensory-evoked potentials and central motor conduction time [5]. In addition, functional MRI studies showed normal activation of the cortex adjacent to enlarged PVS [6–8]. Only three case studies of patients with widespread enlarged PVS in the cerebral white matter who developed neurological symptoms have been previously reported [2–4]. In one case, a patient with bilateral lesions of enlarged PVS developed dementia [2], while a second patient developed transient encephalopathy triggered by a mild respiratory infection [3]. The third case developed dementia and focal signs, where posterior and mediotemporal cerebral lesions were associated with hemiparesis and homonymous hemianopsia [4]. Enlarged PVS have been also correlated with poorer cognitive functions [9]. These findings are in line with our case report. Indeed, our patient initially showed no apparent neurological deficits. However, upon respiratory infection, she developed dementia and focal neurological deficits, including fluent aphasia, agraphia, acalculia, and left-right disorientation. Considering the broad perilesional gliosis, we assumed that there was a latent brain parenchymal damage caused by perivascular fluids pressure and inflammation due to enlarged PVS [14, 15]. Subsequently, the high fever triggered brain dysfunction, which consequently resulted in neurological symptoms. Fluent aphasia, agraphia, acalculia, and left-right disorientation might be associated with enlarged PVS in the left temporal hemisphere. The hypoperfusion and slow wave sites evidenced by SPECT and EEG, respectively, were associated with cystic lesions and indicated that widespread enlarged PVS impaired focal brain functions.

Although the underlying mechanisms of enlarged PVS have yet to be elucidated, recent studies revealed that the pulsatile components of blood pressure, namely the systolic blood pressure and pulse pressure, are associated with enlarged PVS [15]. However, previous studies have mainly focused on small-sized PVS (i.e., cerebral lacunae type III-a according to Poirier’s classification [16]), and indicated that small PVS are biomarkers of vascular risk [15, 17–19]. In contrast, our case presented mainly multiple large PVS occupying the brain parenchyma (i.e.; cerebral lacunae type III-d according to Poirier’s classification [16]), with no evidence of vascular risk factors. Thus, we considered that the enlarged PVS may have resulted from a dysfunctional drainage pathway through the PVS rather than from the continuous high pulsatility to penetrating arteries.

One of the central PVS functions is their contribution to the fluid movement and drainage in the brain. In particular, PVS are thought to be an important drainage pathway between the CSF and brain parenchyma [20–22]. Previous animal studies have demonstrated that the brain interstitial fluid is cleared into the CSF along the perivascular pathway. Therefore, decreased clearance of interstitial solutes occurs when the perivascular pathway is disrupted [20, 21]. Hence, widespread enlarged PVS may lead to brain dysfunction.

In conclusion, our case indicated that widespread enlarged PVS could be a potential cause of neurological deficits. We propose that the pathogenesis of widespread enlarged PVS might occur at the level of perivascular circulation.

## Abbreviations

123I-IMP-SPECT: N-isopropyl-[123I] p-iodoamphetamine-single photon emission computed tomography
CSF: Cerebrospinal-fluid
CT: Computed tomography
EEG: Electroencephalography
MMSE: Mini-Mental State Examination
MRI: Magnetic resonance imaging
PVS: Perivascular spaces
